# The double-edged sword of personality in shaping craftsmanship spirit: an investigation of conscientiousness and openness to experience

**DOI:** 10.3389/fpsyg.2024.1332257

**Published:** 2024-01-31

**Authors:** Zhi Li, Fangmei Lu, Gang He

**Affiliations:** ^1^Business School, Nanjing University, Nanjing, China; ^2^Guanghua School of Management, Faculty of Economics and Management, Peking University, Beijing, China

**Keywords:** conscientiousness, openness to experience, craftsmanship spirit, latent growth modeling, big five personality

## Abstract

**Background:**

Craftsmanship is associated with various positive outcomes at both individual and organizational level, and thus has attracted scholarly attention on examining its antecedents. While craftsmanship can be shaped by both contextual factors and personal traits, existing research has dominantly focused on the former, leaving the latter less examined. Such a lack of examination limits our understanding of craftsmanship in workplace.

**Objective:**

Following the view that individuals’ intrinsic desire to do the job as the core of craftsmanship, we define *craftsmanship spirit* (CS) as an individual’s psychological state of feeling competent, transcendent, and valuable during work, which evolves as an individual’s skills and knowledge expand. We then draw on the classic dispositional literature to explore how individuals’ personality traits (conscientiousness and openness to experience) shape the development of CS differently (i.e., the initial level and the developmental trajectory), and test our theory using a latent growth modeling (LGM) approach.

**Methods:**

We conducted a four-round on-site questionnaire survey with participants who were employees at a large manufacturing company in China. The final sample consists of 746 matched respondents. Data analysis was performed in Mplus 8.3.

**Results:**

Empirical results confirm our hypotheses that both conscientiousness and openness to experience have a positive effect on the initial level of CS. Besides, conscientiousness has a negative effect on the subsequent growth of CS. However, the proposed negative effect of openness to experience on the subsequent growth of CS was not supported when the other four personality traits were considered simultaneously.

**Conclusion:**

This study reveals that conscientiousness and openness to experience have an important effect on CS. Specifically, both conscientiousness and openness to experience are associated with a high level of CS, and the former is associated with low growth of CS. This study not only broadened our understanding on the antecedents of CS, but also provided a dynamic perspective to understand CS in workplace.

## 1 Introduction

In the contemporary world, where specialization and creativity play pivotal roles, craftsmanship spirit, or the pursuit of transcendence and excellence, is increasingly gaining prominence. It is well established that craftsmanship spirit is strongly associated with the creation of top-tier products and services (Halldorsson, 2017; Wu, 2022). Duan et al. (2021) shows that craftsmanship spirit is associated with high organizational resilience. It is also found that craftsmanship has positive effect on several individual outcomes, including personal initiative (Gao, 2022), well-being (Li et al., 2021), and career success (Xue et al., 2022). Given the importance of craftmanship spirit, scholars naturally generate interest in exploring its antecedents.

Craftsmanship spirit, as a type of psychological state, can be influenced by both contextual factors and personal trait (Barrick et al., 2013). However, existing research have dominantly focused on how individuals’ work environment (e.g., organizational learning, leader characteristics) shaped craftsmanship (Deng and Xiao, 2020; Chen et al., 2022; Zhu et al., 2022), leaving how individual factors may shape craftsmanship spirit less examined. This ignorance is problematic because CS is often associated with repetitive and time-consuming tasks, leading people view it as a sacrifice of personal freedom and happiness for the sake of creating material wealth. This implies that individuals’ drives may be important factors that shape CS. However, our knowledge of how individuals’ personal traits matter is limited.

To fill this gap, we draw on the classic dispositional literature to explore the influence of individuals’ personality traits on the development of craftsmanship spirit. The Big Five framework is universally applicable across different cultural backgrounds (Benet-Martinez and John, 1998) and relatively stable over time (Judge et al., 1999). Research has indicated that conscientiousness and openness to experience are essential predictors of work-related variables such as career pursuits, self-realization, and job responsibilities (Holland, 1985; George and Zhou, 2001; Woods and Hampson, 2005). Such relevance motivates us to investigate how conscientiousness and openness to experience shape the development of CS. Specifically, we propose that either a high level of conscientiousness or openness to experience is associated with a high level of the initial state of craftsmanship spirit (I-CS), both of which, however, may lead to slow growth of craftsmanship spirit (G-CS). We adopted a latent growth modeling (LGM) to test our propositions. Empirical results using data collected from a four-wave on-site questionnaire survey with a final sample of 746 respondents, provide support for our major arguments.

Our study makes two major contributions. First, our study reveals that conscientiousness and openness to experience are two important antecedents of CS, thereby broadening our understanding of which personal traits matter in the development of CS. Second, our study supplements a dynamic perspective to understand craftsmanship spirit in workplace. This is important given that psychological states are dynamically changing (Gagné, 2014; Kleine et al., 2019; Tang and Vandenberghe, 2020), but previous studies on craftsmanship spirit largely adopt a static perspective (Thorlindsson et al., 2018; Deng and Xiao, 2020; Gao, 2022). Using latent growth modeling, our study shows *how* conscientiousness and openness to experience shape the development of the craftsmanship spirit in different ways over time.

## 2 Theoretical development

### 2.1 Craftsmanship spirit

Traditionally, craftsmanship spirit refers to the exquisite handiwork of traditional artisans whose livelihood primarily depends on manual skills. Today, craftsmanship spirit encompasses a broader understanding of meticulous workmanship and attention to detail, regardless of the tools used. Scholars have studied the connotation of craftsmanship spirit in different industries and occupations. For example, in the context of the police force, craftsmanship emphasizes apprenticeship, a well-rounded understanding of maintaining law and order rather than task-oriented expertise, a lack of fear of authority, and a focus on oral tradition rather than written documentation (Crank, 1990). For nurses, craftsmanship involves the voluntary and disciplined application of learned skills, the analysis of outcomes, making decisions based on this analysis, and ongoing learning and innovation (Meal and Timmons, 2012). In management sciences, “academic craftsmanship” refers to “the noble and socially responsible pursuit of perfection in creating new understandings about the world of organizations” (Baer and Shaw, 2017, p. 1214).

Sennett (2008) views craftsmanship as the desire to do something well for its own sake, as a basic and enduring human impulse (p. 9). Pratt et al. (2013) and Thorlindsson et al. (2018) also note that individuals’ intrinsic desire to do the job is the core of craftsmanship. In other words, individuals’ subjective experience of transcendence and excellence when doing job is the key to their long-term persistence in striving for excellence. Following this view, we define *craftsmanship spirit* (CS)^1^ as an individual’s psychological state of feeling competent (I can do it), transcendent (I’m making progress), and valuable (I’m making contributions) during work (Gao et al., 2020; Liu et al., 2022); this state evolves as an individual’s skills and knowledge expand.

Craftsmanship spirit, as a type of psychological state, can be influenced by both contextual factors and personal traits (Barrick et al., 2013). However, existing research have dominantly focused on how individuals’ work environment (e.g., organizational learning, leader characteristics) shaped craftsmanship. For example, Chen et al. (2022) reveals a positive effect of organizational learning on employees’ craftsmanship spirit. It is also found that spiritual leadership (Zhu et al., 2022) and humble leadership (Deng and Xiao, 2020) positive influence employees’ craftsmanship spirit. At the individual level, Xue et al. (2022) reveals a positive effect of individual psychological resilience and craftsmanship spirit. After reviewing recent studies on craftsmanship spirit, Gao et al. (2020) calls for future investigations on the influence of individuals’ personality traits on craftsmanship spirit. In response to this call, we draw on Big Five Theory to explore the influence of personality traits on craftsmanship spirit.

### 2.2 Personality and craftsmanship spirit

The Big Five Theory provides a comprehensive theoretical framework for characterizing an individual’s patterns of thought, emotion, and behavior. There are five fundamental dimensions: conscientiousness, openness to experience, extraversion, agreeableness, and emotional stability (neuroticism). This framework is based on four basic assumptions about human nature (McCrae and Costa, 1996a,b): knowability, rationality, variability and proactivity. Proactivity assumption refers to that the causes of human behavior should be sought in the individual. Big-Five implies that personality traits actively and interactively shape people’s lives, and understanding the origins of behavior or feelings from a personality perspective is valuable and necessary (Soldz and Vaillant, 1999).

Within the framework of Big Five Theory, personalities are regarded as deeper psychological entities that can only be inferred from behavior and personal experiences (McCrae and John, 1992). Intrinsic psychological states and interpersonal features serve as expressions of these fundamental tendencies. They evolve over time in response to physiological maturation, changes in social roles, shifts in external expectations, or deliberate interventions. These expressions are described as “characteristic adaptations.” Craftsmanship spirit can be seen as an expression of these fundamental tendencies.

This study places particular emphasis on the influence of conscientiousness and openness to experience on craftsmanship spirit. Research indicates that conscientiousness and openness to experience can predict career interests and choices (Holland, 1985; Woods and Hampson, 2005). They serve as essential predictors for work-related variables such as performance and creativity and are closely associated with career pursuits, self-realization, and job responsibilities (George and Zhou, 2001). And management scholars are concerned with the realization of craftsmanship spirit in organizations, that is, in the workplace. This kind of psychological state is achieved through interactions with work. In comparison to conscientiousness and openness to experience, extraversion, agreeableness, and emotional stability do not have direct and close relationships with labor at work. Although they were not the primary predictors that we aimed to examine, we included them as controls in our model.

#### 2.2.1 Conscientiousness and craftsmanship spirit

Conscientiousness is one of the dimensions within the Big Five personality framework (Digman, 1990). It primarily characterizes an individual’s drive for achievement, organizational planning, resilience, self-control, acceptance of traditional norms, as well as virtues and responsibilities toward others (Barrick et al., 2002; Roberts et al., 2005; Zhao et al., 2010; Hough et al., 2015). Conscientiousness can be further divided into two dimensions: duty and achievement (Tangirala et al., 2013). Duty, also referred to as reliability (Moon, 2001), is typically other-orientated, reflecting a belief in emphasizing one’s mission and duty toward society and others (Becker, 1998). Highly conscientious individuals tend to assist others because they genuinely care about the well-being of others (Moon et al., 2008). Achievement orientation (Dudley et al., 2006) is self-directed and reflects an individual’s pursuit and aspiration for progress and achievement in their career (Chae et al., 2019).

Conscientiousness is a trait that serves as a driving force, continually propelling individuals forward in their pursuit of personal growth and self-improvement (Judge et al., 2009). On one hand, from the perspective of the conservation of resources theory (Hobfoll, 2001), high conscientiousness represents a dispositional resource (Zellars et al., 2006; Judge and Zapata, 2015). Craftsmanship spirit, as a psychological state, does not spontaneously emerge in the workplace but rather develops through actively engaging in work. Individuals with high levels of conscientiousness, as compared to those with low levels, are motivated to continuously acquire knowledge and master the skills necessary for their work. They are driven to expand and enhance their personal and professional resources for future career development (Ocampo et al., 2020). This set of characteristics represents valuable resources in the workplace, enabling individuals to complete their work to a high standard and make ongoing improvements. Considering the two dimensions of conscientiousness, duty orientation encourages individuals to focus on whether they are accountable to others and the organization in their work, as well as whether they are creating value for society development. Achievement orientation, on the other hand, prompts individuals to equip themselves for their career development and achievements, enhancing their job performance to ensure that their skills remain up-to-date, meeting their competence and transcendence needs.

On the other hand, individuals with high conscientiousness tend to exhibit higher goal commitment (Barrick et al., 1993). Those with high conscientiousness display a greater level of commitment to exert effort in achieving both work-related and personal goals (Sutin and Costa, 2010). They excel in self-restraint and self-motivation, subsequently fulfilling their job responsibilities (Barrick et al., 2002). High conscientious individuals set higher performance goals and efficiently allocate resources to achieve these goals (Halbesleben et al., 2009). Once they set their goals, highly conscientious individuals engage in processes such as considering the essence of their work, acquiring necessary skills, mobilizing all available resources, referencing successful cases, and overcoming various challenges to achieve their goals. This process helps them attain the psychological state of craftsmanship spirit.

In summary, this study posits that individuals with high conscientiousness are more likely to feel competent, transcendent, and valuable in their work. This personality trait serves as an endowment resource, exerting a positive influence on the initial state of craftsmanship spirit. Therefore, we propose the following hypothesis:

*Hypothesis 1*: Conscientiousness is positively related to craftsmanship spirit initial state (intercept factor).

For the journey (change) of craftsmanship spirit, conscientiousness may have negative effects. First, individuals with high conscientiousness tend to be self-critical and pursue perfection (Hill et al., 1997; Dunkley et al., 2006), making them more susceptible to stress from negative feedback (Cianci et al., 2010). Highly conscientious individuals have high personal standards, are disciplined, and prefer to be in control. Negative feedback can make them feel “out of control.” Although highly conscientious individuals work diligently and conscientiously, as their work abilities and performance are already at a relatively high level, over time, the incremental gains in self-transcendence and a sense of value in their work are smaller. This can easily lead to a sense of “discontent,” negatively affecting changes in craftsmanship spirit, with slower increases in sense of competence, transcendence, and value. So conscientiousness, as a personality resource and endowment, not only contributes to a high initial state but also exerts an inhibitory effect on the growth of craftsmanship spirit.

Second, when an individual’s conscientiousness is high, they may procrastinate due to excessive attention to detail and the pursuit of perfection or become stuck due to overthinking, causing tasks to stall (Widiger et al., 2002; Samuel and Widiger, 2011). Procrastination and stagnation inevitably lead to work pause, or even internal consumption, resulting in a slower increase in craftsmanship spirit.

Third, based on the assumption of a positive relationship between conscientiousness and the initial state of craftsmanship spirit, responsible individuals are often self-driven, and their job performance is excellent. Therefore, making improvements at work on this basis is often more challenging and requires creative breakthroughs. However, research shows that in turbulent situations, individuals with high conscientiousness adapt less effectively than those with low conscientiousness, as the reliability dimension of conscientiousness plays a major role and to some extent limits an individual’s decisiveness (LePine et al., 2000). Therefore, we propose the following hypothesis:

*Hypothesis 2*: Conscientiousness is negatively related to the growth rate of craftsmanship spirit (slope factor).

#### 2.2.2 Openness to experience and craftsmanship spirit

Openness to experience is another important dimension of the Big Five personality traits. It focuses on an individual’s curiosity, creativity, and imagination. Additionally, individuals with high openness to experience seek novelty (De Fruyt et al., 2000), are enthusiastic about exploring new ideas and viewpoints, and seek diverse values and esthetic standards (Zhao et al., 2010). We propose that openness to experience has a positive effect on the initial state of craftsmanship spirit for three reasons.

First, from an information processing perspective, highly open individuals exhibit a greater capacity and inclination to seek, identify, understand, and utilize more information than those with lower levels of openness (DeYoung et al., 2014). They further connect and integrate this information with existing knowledge, creatively solving work-related problems (Jach and Smillie, 2021). In this process, highly open individuals unleash their intelligence, enjoying the use of knowledge and information to solve problems, effectively managing the progress of their work. In other words, their wisdom is crucially utilized. In terms of information itself, open individuals tend to prefer abstract and theoretical information, whereas closed individuals favor concrete and tangible information (Boyle et al., 2008). Abstract information often provides greater room for interpretation and exploration, offering more possibilities. For example, when considering new employees entering an organization, open individuals tend to explore the spatial layout of the organization, observe how different departments collaborate, actively engage in discussions with colleagues they can connect with, and identify critical aspects of the work. Improving and striving for excellence are at the core of craftsmanship spirit. Enhancing and transcending the current state is not easy and requires deep thinking and innovation.

Secondly, from the perspective of work motivation, open individuals appreciate the freedom to work for themselves (Roccas et al., 2002). Openness is a significant predictor of vocational interests (Costa et al., 1984), especially with intellectual curiosity being a more important predictor of vocational interests (Costa et al., 1995). They lean toward tasks that require personal initiative, such as jobs that offer autonomy and diversity, as well as growth opportunities (Bipp, 2010). Being open also implies being sensitive and self-aware. Open individuals are sensitive to their innermost feelings, pursuing esthetic and pleasurable aspects of their work. This, in itself, motivates individuals to refine and improve their work. They tend to focus on improving the finer details of their work, ensuring that their work is not only of high quality in terms of tangible output but also in terms of their personal feelings. This is related to the self-cosmic view of open individuals, who enjoy contemplating their relationship with the universe and their essence. Highly open individuals tend to derive gains from their own existence in the world, accomplishing self-transcendence through experiences and exploration. Actively exploring ways to enhance their work is crucial as craftsmanship spirit is not about blindly repeating tasks but sincerely delighting in achieving higher-quality work. Open individuals approach new technologies and methods with openness and eagerness, valuing the potential of new elements to enhance their past work. In contrast, those with low openness may be more content with the status quo, preferring tried-and-true methods that reduce uncertainty.

Thirdly, highly open individuals display greater adaptability and are more open to different job requirements and changing circumstances. This adaptability is essential for promoting craftsmanship spirit in the context of the modern era. Craftsmanship spirit is not synonymous with an unchanging, purely manual approach. In interviews with master craftsmen, they often mention phrases like “*we need to utilize new tools and methods, incorporating modern aesthetic elements into our creations*,” and “*meticulous improvements originate in the mind*.”^2^ Open individuals make better decisions and adapt more effectively in a changing environment, efficiently adjusting to unexpected changes (LePine et al., 2000). On one hand, open individuals welcome new elements, so change is not a threat but an opportunity for them to make improvement. They believe in the principle of “*without destruction there can be no construction*.”^3^ Conversely, individuals with low levels of openness experience significant anxiety when their stable and familiar environment is disrupted, leading to a state of passivity and confusion. On the other hand, highly open individuals are more inclined to engage in self-examination and evaluation, which is necessary for learning in a changing environment (Busato et al., 1999). They are adept at learning and restructuring existing cognitive structures during change, leading to self-improvement.

In conclusion, this paper proposes that individuals with high openness to experience are better equipped for deep information processing, creative problem-solving, and choosing work guided by their own feelings and interests. They also exhibit strong adaptability and resilience. These qualities are part of an ongoing process of self-reflection and self-transcendence, and have a positive impact on the initial state of craftsmanship spirit. Therefore, we propose the following hypothesis:

*Hypothesis 3*: Openness to experience is positively related to the initial state of craftsmanship spirit (intercept factor).

When it comes to the impact of openness to experience on the change of craftsmanship spirit, its effects can potentially be negative. Individuals with high openness to experience may have a relatively higher initial state of craftsmanship spirit, their subsequent growth of craftsmanship spirit tend to be slower for two reasons.

First, to achieve higher levels of craftsmanship spirit, such as reaching an exceptional state, often requires a deep dive into a particular field or industry and continuous dedication. From a career development perspective, the desire for new opportunities and challenges is not always conducive to maintaining focus and sustained effort in a single job or profession (Ciavarella et al., 2004). High openness individuals may be more inclined to explore new career paths, which can lead to more frequent job changes. While they may be more committed in the early stages of a job, they are quick to become disenchanted if they do not find meaning and value, leading to the exploration of new career paths and a state of diversified development, potentially exhibiting a “short-lived” interest. Headey and Wearing (1989) found that open individuals experience more life events, whether positive or negative. Open individuals are committed to finding their true calling, something that aligns with their self and provides a sense of meaning and value in their lives. In the absence of finding it, they tend to divert their attention to a wider range of activities, such as participating more frequently in cultural activities that require their contribution of ideas (Schwaba et al., 2018).

Second, individuals may become entrenched in self-centered self-improvement (Dunlop et al., 2017). Because openness is closely related to curiosity and intelligence, open individuals tend to perceive their own intellectual capabilities and engage in intellectual activities (DeYoung et al., 2007). They are highly attuned to subjective experiences and the depth of their thinking, which may lead to detachment from reality, a focus on conceptual thinking, and a reluctance to take action, remaining in the realm of contemplating meaning and value. In extreme cases, this can lead to feelings of depression.

Taking together, individuals with high openness may pursue diversified career paths or become overly focused on their subjective experiences, remaining disconnected from the practical aspects and actions. These, in turn, hinder the further development of craftsmanship spirit. We thus propose the following hypothesis:

*Hypothesis 4*: Openness to experience is negatively related to the growth rate of craftsmanship spirit (slope factor).

## 3 Materials and methods

### 3.1 Participants and procedure

We utilized an onsite survey study to test the model. Participants were employees from a manufacturing company in Nanjing, China. Before formal data collection, we explained the purpose of our study to the Human Resources Manager. With the help of staff from the HR Department, we distributed questionnaires to all employees at various departments including the manufacturing department, the finance department, the distribution department, quality management department, and the human resources management department. To reduce potential biases induced by common methods (Podsakoff et al., 2003), we collected data at four time points with 1 month time intervals. At time 1, participants provided data on their big five personality and craftsmanship spirit (response rate = 100%, *N* = 812). During time 2–4, participants provided data on craftsmanship spirit (*N* = 746 at Time 4, response rate = 100%). During the data collection process, we assured participants that their answers were confidential, and delivered a small gift to each participant before administering the surveys. Participants’ demographic information was provided by the HR Department at time 1. By including matched data only, the final sample consist of 746 respondents. MANOVA analysis revealed that there were no significant systematic differences (Wilks’ lambda = 0.99, *p* > 0.05) between our respondents and non-respondents in term of conscientiousness and openness to experience.

### 3.2 Measures

Measures developed in English were converted into Chinese following the translation and back-translation procedure recommended by Brislin (1980). A 6-point Likert scale was used for item rating, with response options ranging from 1 (*strongly disagree*) to 6 (*strongly agree*).

Conscientiousness. Conscientiousness was measured using the 12-item scale developed by Costa and McCrae (1992). Sample items are “I always accomplish my task carefully,” “I have a series of specifically and definitely goals,” “I will accomplish them step by step,” and “I always try my best to do things perfectly” (Cronbach’s α = 0.95). To make it easier for participants to understand, we have adjusted the negatively scored items to positively scored ones (All measurements in this study are positively scored).

Openness to Experience. Openness to experience was measured using the 12-item scale developed by Costa and McCrae (1992). Sample items are “I am very interested in the manifestation of art and nature,” “I have no interest in thinking over the nature of human being or the universe,” and “I usually like using theoretical and abstract concept” (Cronbach’s α = 0.91).

Craftsmanship Spirit. Craftsmanship spirit was measured using a 9-item scale adapted from Liu et al. (2022). Participations were asked to evaluate their craftsmanship spirit over the past month of work. Sample items are “I possess sufficient knowledge and skills,” “I am competent in my job,” “I work hard and make contributions in my work, realizing my personal value,” and “Exploring new ideas and methods at work makes me feel like I am continuously growing.” Cronbach’s α of T1-T4 were 0.94, 0.95, 0.95, 0.96, respectively. The full scale can be found in the Appendix.

Control Variables. Gender, length of service (in terms of employment tenure), education, and another three personalities were controlled. Information on gender, tenure, and education was provided by HR department. Gender was a dummy variable (0 = female,1 = male). Length of service is measured in years, from the time of entering the workforce. Education was measured in six intervals from 1 (junior high school diploma) to 6 (Master). Extroversion, agreeableness, and emotional stability were measured using the scale developed by Costa and McCrae (1992). Emotional stability is reverse-scored from the neuroticism score. Due to the high correlation between agreeableness and conscientiousness measurements, although they are theoretically distinct concepts, to reduce multicollinearity, we retained four items to measure agreeableness (Eight items were removed based on the correlation between conscientiousness and agreeableness measurement items, as well as the results of confirmatory factor analysis, Cronbach’s α = 0.84). Extroversion (Cronbach’s α = 0.94) and emotional stability (Cronbach’s α = 0.95) are each measured by 12 items.

### 3.3 Analyses

We conducted data analysis using a latent growth modeling (LGM) approach (Bollen and Curran, 2006; McArdle, 2009) in Mplus. While a two-point analysis can determine a straight line, it takes three or more points to observe changes within individuals (Trougakos et al., 2014). The LGM approach allows researchers to analyze variables that change over time, revealing the developmental trends of these variables. For craftsmanship spirit, the intercept and the slope, were specified from four repeated measures. The intercept represents the relative initial level of craftsmanship spirit, while the slope indicates the growth rate (trajectory) of craftsmanship spirit. In our study, the intercept was located at the initial measurement time (Time 1), by fixing all the intercept factor loadings at one, and the first slope factor loading to 0.

## 4 Results

### 4.1 Preliminary analyses

Table 1 shows mean values, standard deviations, correlations of study variables.

**Table 1:** Descriptive statistics.

Vars	Mean	sd	1	2	3	4	5	6	7	8	9	10	11
1. Gender	0.51	0.50	1										
2. LoS	8.20	7.13	−0.20^***^	1									
3. Edu	2.55	1.24	0.01	0.39^***^	1								
4. Con	5.14	0.68	−0.04	−0.04	−0.12^**^	1							
5. Oe	4.54	0.85	−0.03	−0.04	−0.07^*^	0.58^***^	1						
6. Ex	4.77	0.86	−0.01	−0.07	−0.15^***^	0.58^***^	0.67^***^	1					
7. Ag	4.92	0.85	−0.01	−0.01	−0.08^*^	0.69^***^	0.57^***^	0.52^***^	1				
8. ES	3.59	1.32	0.02	0.06	0.10^**^	0.00	−0.31^***^	−0.14^***^	−0.11^**^	1			
9. CS-T1	4.99	0.77	0.00	−0.05	−0.12^**^	0.67^***^	0.53^***^	0.54^***^	0.53^***^	−0.04	1		
10. CS-T2	4.91	0.79	−0.03	0.04	−0.01	0.44^***^	0.35^***^	0.40^***^	0.36^***^	−0.02	0.49^***^	1	
11. CS-T3	4.92	0.78	−0.03	0.06	0.01	0.40^***^	0.34^***^	0.34^***^	0.33^***^	−0.02	0.44^***^	0.47^***^	1
12. CS-T4	4.94	0.77	0.08^*^	−0.02	−0.05	0.36^***^	0.28^***^	0.33^***^	0.29^***^	0.07	0.42^***^	0.43^***^	0.51^***^

*N* = 746; LoS, length of service; Con, conscientiousness; Oe, openness to experience; Ex, extraversion; Ag, agreeableness; ES, emotional stability; C-T*, craftsmanship spirit-Time *; *p < 0.05, **p < 0.01, ***p < 0.001; two-tailed.

**Figure 1:**
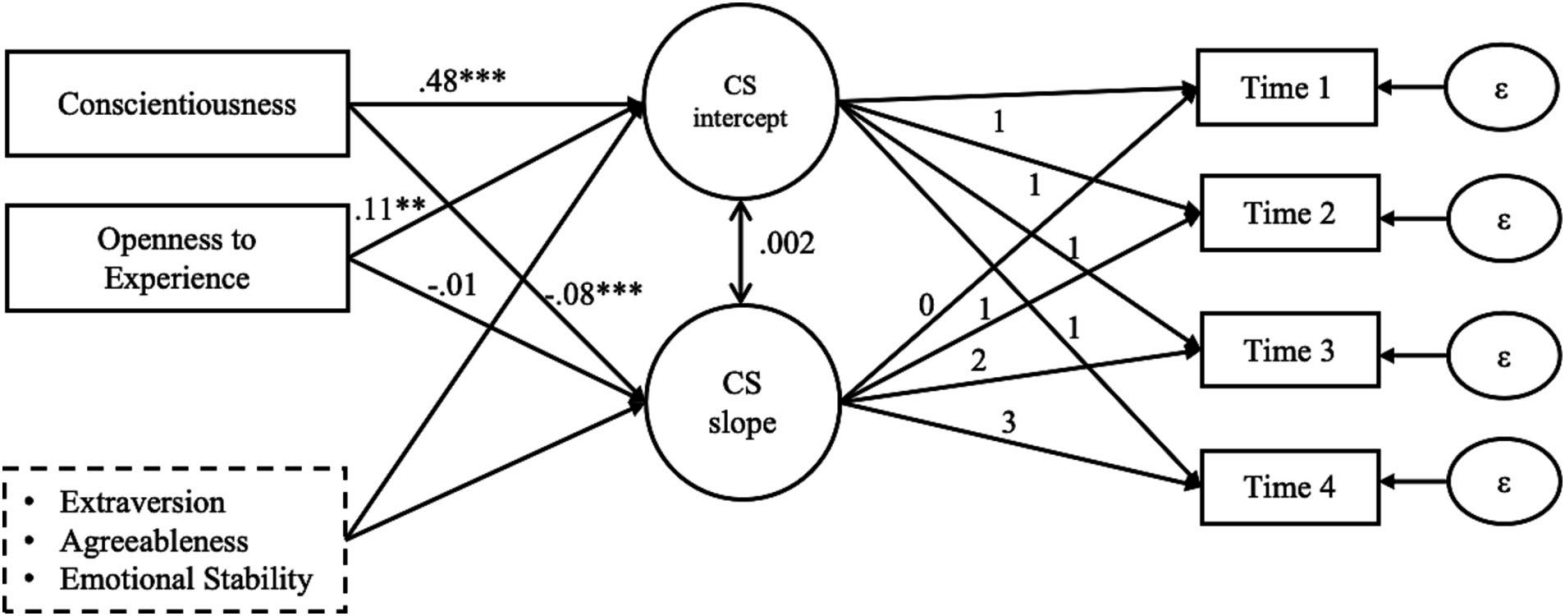
Parameter estimates in the testing model. *N =* 746; CS = craftsmanship spirit; ***p < 0.001, **p < 0.01, *p < 0.05; two-tailed.

Table 2 presents the results of the confirmatory factor analysis (CFA). We conducted CFA separately for each round of craftsmanship spirit measurement and the Big Five personality traits to examine the fit indices of the six-factor model. Due to the relatively large number of items in the personality measures, we employed parceling techniques (Landis et al., 2000). Except for Agreeableness, the 12 measurement items for each of the other four personality traits were parceled into four parcels, and the nine measurement items for craftsmanship spirit were parceled into three parcels. In total, this resulted in a six-factor model with 23 items. As shown in Figure 1, the *p*-values for the chi-squared test were all below 0.05, indicating a good fit between the model and the data. The Comparative Fit Index (CFI) and Tucker-Lewis Index (TLI) values ranged from 0.90 to 1.00, suggesting a good model fit. The standardized root mean square residual (SRMR) and the root mean square of error of approximation (RMSEA) fell between 0.05 and 0.08, with some values below 0.05, indicating a reasonable or good model fit (Browne and Cudeck, 1993; Kline, 2015). Taken together, these results support a high level of discriminant validity for our measures.

**Table 2:** Model fit statistics for testing discriminant validities and measurement invariance.

Model/variable	*χ*2	DF	CFI	TLI	RMSEA	SRMR
**Measurement model (six factors: big five personality, craftsmanship spirit)**						
Measurement model T1	781.812^***^	215	0.965	0.959	0.059	0.03
Measurement model T2	749.128^***^	215	0.966	0.961	0.058	0.029
Measurement model T3	724.201^***^	215	0.968	0.963	0.056	0.029
Measurement model T4	697.046^***^	215	0.970	0.965	0.055	0.028
**Longitudinal measurement invariance across four waves**						
Configural invariance	282.514^***^	48	0.968	0.956	0.081	0.027
Metric/weak invariance	319.427^***^	54	0.964	0.956	0.081	0.061
Scalar/strong invariance	341.639^***^	63	0.962	0.961	0.077	0.067
Error variance /strict invariance	421.47^***^	72	0.953	0.957	0.081	0.053

DF, degree of freedom; CFI, comparative fit index; TLI, Tucker-Lewis index; RMSEA, root mean square error of approximation; SRMR, standardized root mean square residual; ^***^p < 0.001.

Furthermore, to conduct LGM analysis, it is essential to ensure measurement invariance (Chan, 1998). We examined the measurement invariance of repeated craftsmanship spirit measures. Results presented in Table 2 indicated that configural invariance, metric (weak invariance), scalar (strong invariance) and error variance (strict invariance) received reasonable fit to the data. These results show sufficient measurement invariance across the 4-month study span.

### 4.2 Hypotheses tests

Results from the LGM were presented in Table 3. In Model 1, only demographic variables were included. Model 2 further included extraversion, agreeableness, and emotional stability as controls. Conscientiousness and openness to experience were included in Model 3. Figure 1 displays the results of Model 3.

**Table 3:** Estimated path coefficients for latent growth models.

Predictors	Model 1 CS		Model 2 CS		Model 3 CS		Model 4 CS	
	Intercept	Slope	Intercept	Slope	Intercept	Slope	Intercept	Slope
Gender	−0.03 (0.05)	0.03 (0.02)	−0.02 (0.04)	0.18 (0.02)	0.01 (0.04)	0.02 (0.02)	−0.01 (0.05)	0.03 (0.02)
Length of service	0.00 (0.00)	0.00 (0.00)	0.00 (0.00)	0.01 (0.00)	0.00 (0.00)	0.00 (0.00)	0.00 (0.00)	0.00 (0.00)
Edu	−0.05^*^ (0.02)	0.01 (0.01)	−0.01 (0.02)	0.06^***^ (0.01)	−0.00 (0.02)	0.01 (0.01)	−0.03 (0.02)	0.01 (0.01)
Extraversion			0.53^***^ (0.03)	−0.22^*^ (0.01)	0.14^***^ (0.04)	−0.00 (0.02)		
Agreeableness			0.50^***^ (0.03)	−0.35^***^ (0.01)	0.06 (0.03)	−0.01 (0.02)		
Emotional stability			0.04 (0.02)	0.07 (0.01)	0.01 (0.03)	0.01 (0.02)		
Conscientiousness					0.48^***^ (0.03)	−0.08^***^ (0.02)		
Openness to experience					0.11^**^ (0.02)	−0.01 (0.01)	0.45^***^ (0.03)	−0.07^***^ (0.01)

*N* = 746; CS, craftsmanship spirit; ***p < 0.001, **p < 0.01, *p < 0.05; two-tailed; The data in parentheses represent the standard errors.

Hypothesis 1 predicts that conscientiousness is positively related to the intercept of craftsmanship spirit. As shown in Figure 1, there is a significant positive relationship between conscientiousness and the intercept of craftsmanship spirit (β = 0.48, *p* < 0.001), indicating that individuals with higher conscientiousness have higher initial level of craftsmanship spirit. Therefore, hypothesis 1 is supported.

Hypothesis 2 predicts that conscientiousness is negatively related to the slope of craftsmanship spirit. Figure 1 shows a significant negative relationship between conscientiousness and the slope of craftsmanship spirit (β = −0.08, *p* < 0.001), indicating that conscientiousness appears to slow down the growth rate of craftsmanship spirit. Hypothesis 2 is supported.

Hypothesis 3 suggests that openness to experience is positively related to the intercept of craftsmanship spirit. Figure 1 shows a significant positive relationship between openness to experience and the intercept of craftsmanship spirit (β = 0.11, *p* < 0.01), indicating that individuals with higher openness to experience have higher initial level of craftsmanship spirit. Hence, hypothesis 3 is supported.

Hypothesis 4 suggests that openness to experience is negatively related to the slope of craftsmanship spirit. Figure 1 shows that the relationship between openness to experience and the slope of craftsmanship spirit is negative (β = −0.01) but not significant (*p* > 0.1). Hypothesis 4 is not supported. One reason for this finding could be that conscientiousness has a stronger effect than openness to experience, the simultaneous consideration of both traits weakens the effect of openness to experience. We thus conducted a supplementary analysis to examine the independent effect of openness to experience, results were presented in Model 4 of Table 3. Model 4 shows that the effect of openness to experience on the intercept of craftsmanship spirit is significantly positive (β = 0.45, *p* < 0.001), this is consistent with hypothesis 3; and the effect of openness to experience on the slope of craftsmanship spirit is negative and significant (β = −0.07, *p* < 0.001), which is consistent with the prediction of hypothesis 4. Taken together, these results support our suggestion that conscientiousness has a stronger influence on the growth rate of craftsmanship spirit than openness to experience.

Furthermore, results in Model 3 show that extraversion has a significantly positive effect on the intercept of craftsmanship spirit (β = 0.14, *p* < 0.001). Extraversion is closely linked to social interactions, and individuals with high extraversion tend to have more social relationships and social support (Berkman et al., 2000). They are more likely to establish connections with colleagues, superiors, and clients, which enables them to gain feedback, or build collaborations. They are also more likely to maintain a positive emotional state, possess abundant energy, and utilize their proactivity and initiative to achieve goals. Thus, extraversion may have a positive influence on the initial level of craftsmanship spirit.

## 5 Discussion

### 5.1 Conclusion

Craftsmanship spirit, as an individual’s psychological state of feeling competent, transcendent, and valuable during work, is important in contemporary workplace. However, our knowledge of individuals’ personal traits that shape craftsmanship spirit is limited. Drawing on the big five personality framework, this study examines the impact of conscientiousness and openness to experience on the development of craftsmanship spirit. Results from LGM confirm that both conscientiousness and openness to experience positively influence the initial level of craftsmanship spirit; and conscientiousness negatively influence the subsequent growth of craftsmanship spirit.

### 5.2 Theoretical contributions

This study makes two major contributions. First, our investigation on the influence of conscientiousness and openness to experience on CS broadens our understanding of its antecedents. Existing studies on the antecedents of craftsmanship spirit play a greater emphasis on situational factors either at the organizational level or at the leader level. For example, at the organizational level, Chen et al. (2022) reveals a positive impact of organizational learning on fostering employee craftsmanship spirit. At the leader level, research shows that spiritual leadership (Zhu et al., 2022) and humble leadership (Deng and Xiao, 2020) are important antecedents of employee craftsmanship spirit. Nevertheless, there is limited investigation on the influence of individuals’ personal traits on craftsmanship spirit. Following the position that personalities are fundamental tendencies serve as a resource endowment impacting craftsmanship spirit, our study reveals that both conscientiousness and openness to experience have a positive effect on the initial level of CS, but conscientiousness has a negative effect on the subsequent growth of CS. Additionally, our empirical results reveal a positive effect of extraversion on the initial level of CS. Our study responses to the recent call for exploring personal antecedents of craftsmanship spirit (Gao et al., 2020).

Second, our study supplements a dynamic perspective to understand craftsmanship spirit in workplace. Craftsmanship spirit, as a psychological state, is dynamically changing (Gagné, 2014; Kleine et al., 2019; Tang and Vandenberghe, 2020). However, prior studies have primarily adopted a static perspective (Thorlindsson et al., 2018; Deng and Xiao, 2020; Gao, 2022). This shift is important and necessary because that a dynamic perspective can help us more comprehensively understand the distinct ways in which personality traits shape the initial level and the development trajectory of craftsmanship spirit. By employing a latent growth modeling, this study shows that conscientiousness has a positive effect on the initial level of CS, but it has a negative effect on the subsequent growth of CS. Openness to experience has a positive effect on the initial level of CS, but no significant effect on the subsequent growth of CS. These findings provide empirical evidence for the conceptualization of craftsmanship spirit as a psychological state which evolves as an individual’s skills and knowledge expand. More importantly, it supplements a dynamic perspective to understand variations among individuals’ craftsmanship spirit. In sum, this study indicates that individuals with high conscientiousness tend to have high level craftsmanship spirit, but they are less likely to make significant improvement on their future craftsmanship spirit.

### 5.3 Management implications

This study has several implications. First, by unpacking how individual personality traits influence craftsmanship spirit, our study provides guidelines for managers to better manage and allocate human resources. Specifically, managers should take individuals’ personality traits into account in person-job fit evaluation. Our study reveals that individuals with either high conscientiousness, openness to experience, or extraversion exhibit high initial level of craftsmanship spirit. When hiring or promoting employees to perform tasks that required continual improvement, managers should consider those people as the top candidates.

Second, managers should take individual personality traits into consideration when training employees for the pursuit of continual improvement and perfection. While individuals with high conscientiousness exhibit high initial level of CS, their subsequent growth on CS will be slow. Thus, for employees with high conscientiousness, training programs should be tailored to provide additional resources and support to help them encounter bottlenecks. Meanwhile, managers should pay attention to provide constructive feedback to employees with high conscientiousness. Constructive feedback can help them engage in self-reflection and self-awareness and thus expand their room of future development on craftsmanship spirit.

Our study also has practical implications for employees. By unpacking the relationships between personality traits and the development of CS, our study offers guidelines for employees to manage their own career development. Employees can assess whether their personality traits align with the occupied or targeted job positions in term of CS, and thus to evaluate whether additional resources or support are needed to do jobs well. This is crucial for highly conscientious individuals who are less likely to experience high growth of craftsmanship spirit. To achieve further growth in craftsmanship spirit, those individuals should proactively seek for new or challenging tasks.

Taken together, we encourage managers to help employees know themselves better in term of big five personality and strive for a shared understanding of job expectations with them. Helping employees understand personalities can help to retain like-minded employees. When work becomes a significant avenue for self-expression and self-fulfillment, employees’ dedication to and improvement in their work naturally occur, leading to a win-win situation for both the organization and its employees.

### 5.4 Limitations and further research directions

While this study utilized longitudinal research, it could not comprehensively examine the various combinations of the Big Five personality traits concerning their correlation and causality. Future research could adopt a configurational approach to gain a more comprehensive understanding of how personality affects both the initial levels and growth rate of craftsmanship spirit.

This study examined the influence of personality on craftsmanship spirit, particularly the effects of conscientiousness and openness to experience. However, it did not consider the interaction of situational factors and personality traits on craftsmanship spirit. For individuals with prominent trait tendencies, excessive situational interventions may not necessarily be beneficial, as there may be substitution effects between the situation and personality. When job tasks require a high level of conscientiousness, those with lower conscientiousness may need to create specific situations to propel themselves forward (Fong and Tosi, 2007).

Moreover, in real work scenarios, we often find people exhibiting extroverted or introverted behaviors at different times, and varying levels of responsibility. For individuals whose traits are not highly pronounced, the activation of specific situations might be crucial. Future research can explore the influence of situational factors based on the trait activation theory (Tett and Burnett, 2003). It is possible to investigate whether craftsmanship spirit, being an expression of an individual’s nature, can be enhanced through activation or interventions, and whether individuals with different personality traits respond differently to such interventions.

In sum, this study provides a preliminary exploration of how personality traits affect craftsmanship spirit, but it has some limitations. There are many potential avenues for future research that can further our understanding of this relationship and how to stimulate craftsmanship spirit in the workplace.

## Data availability statement

The raw data supporting the conclusions of this article will be made available by the authors, without undue reservation.

## Ethics statement

The studies involving humans were approved by The Office of Science and Technology, Nanjing University. The studies were conducted in accordance with the local legislation and institutional requirements. The participants provided their written informed consent to participate in this study.

## Author contributions

ZL: Writing – original draft. FL: Writing – review & editing. GH: Writing – review & editing.

## Footnotes

^1^The term “craftsmanship” is used differently in existing studies. We use “craftsmanship spirit” throughout this paper, referring to our definition of craftsmanship spirit as a type of psychological state.

^2^From China’s “Master Craftsman of a Great Nation” documentary series, Season 1.

^3^This a phrase that is often attributed to Karl Marx, a 19th-century philosopher and political theorist.

## Appendix

Table A1. Scale of craftsmanship spirit.

**Table:**

Over the past month of work, I feel ……	
Competent	I have acquired the knowledge and skills required for my job
	I possess sufficient knowledge and skills, and I am competent in my job
	I have enough knowledge and skills, and I am confident in my work
Transcendent	My work benefits many people
	I work hard and make contributions in my work, realizing my personal value
	My job is not just a means of making a living; it is a career I am willing to dedicate myself to
Valuable	Doing each task well at work makes me feel like I am improving every day
	Striving for excellence in my work makes me feel like I am constantly elevating myself
	Exploring new ideas and methods at work makes me feel like I am continuously growing
