# Supplementary material for: The double-edged sword of personality in shaping craftsmanship spirit: an investigation of conscientiousness and openness to experience
Source: Front Psychol. 2024 Jan 31;15:1332257. doi: 10.3389/fpsyg.2024.1332257 (PMC10866028; doi:10.3389/fpsyg.2024.1332257)
Supplement: Supplementary file 1 [file Data_Sheet_1.PDF]

Table A: ANOVA Test Between Two Groups

Variables	Group	N	Mean	Standard error	95% Conf. interval	Difference	F statistic	*p* value
Gender	1	66	0.62	0.49	[0.50, 0.74]			
	2	746	0.52	0.50	[0.48, 0.55]	0.11	2.69	$p > 0.05$
Length of service	1	66	4.17	7.51	[2.32, 6.02]			
	2	746	8.17	7.12	[7.66, 8.86]	-4.00	18.96	$p < 0.001$
Education	1	66	2.05	1.42	[1.70, 2.39]			
	2	746	2.55	1.24	[2.46, 2.64]	-0.50	9.67	$p < 0.001$
Craftsmanship spirit-T1	1	66	4.80	0.82	[4.60, 5.00]			
	2	746	4.99	0.77	[4.94, 5.05]	-0.19	3.75	$p > 0.05$

Note. Group 1 refers to the lost follow-up population; Group 2 refers the follow-up population.
